# Multidisciplinary breast centres in Germany: a review and update of quality assurance through benchmarking and certification

**DOI:** 10.1007/s00404-011-2212-3

**Published:** 2012-02-08

**Authors:** Markus Wallwiener, Sara Y. Brucker, Diethelm Wallwiener, The Steering Committee

**Affiliations:** 1 Department of Obstetrics and Gynaecology, University of Heidelberg, Voßstraße 9, 69115 Heidelberg, Germany; 2 Department of Obstetrics and Gynaecology, Universitäts-Frauenklinik Tübingen, Calwerstraße 7, 72076 Tübingen, Germany

**Keywords:** Guidelines, Certification, Multidisciplinarity, Treatment optimization, Quality assurance, Benchmarking

## Abstract

**Purpose:**

This review summarizes the rationale for the creation of breast centres and discusses the studies conducted in Germany to obtain proof of principle for a voluntary, external benchmarking programme and proof of concept for third-party dual certification of breast centres and their mandatory quality management systems to the German Cancer Society (DKG) and German Society of Senology (DGS) Requirements of Breast Centres and ISO 9001 or similar. In addition, we report the most recent data on benchmarking and certification of breast centres in Germany.

**Methods:**

Review and summary of pertinent publications. Literature searches to identify additional relevant studies. Updates from the DKG/DGS programmes.

**Results and conclusions:**

Improvements in surrogate parameters as represented by structural and process quality indicators suggest that outcome quality is improving. The voluntary benchmarking programme has gained wide acceptance among DKG/DGS-certified breast centres. This is evidenced by early results from one of the largest studies in multidisciplinary cancer services research, initiated by the DKG and DGS to implement certified breast centres. The goal of establishing a nationwide network of certified breast centres in Germany can be considered largely achieved. Nonetheless the network still needs to be improved, and there is potential for optimization along the chain of care from mammography screening, interventional diagnosis and treatment through to follow-up. Specialization, guideline-concordant procedures as well as certification and recertification of breast centres remain essential to achieve further improvements in quality of breast cancer care and to stabilize and enhance the nationwide provision of high-quality breast cancer care.

## Introduction

Breast cancer continues to be the most common malignancy in women both in Germany and worldwide [1, 2]. In Germany, about 47,500 women were newly diagnosed with breast cancer in 2000 [3]. More recent estimates put the figure at approx. 55,000–58,000 new cases per year [1, 4–6]. Age-standardized incidence of breast cancer remained essentially constant between 2000 and 2006 but is expected to rise due to the introduction of mammographic screening programmes [1]. The average lifetime risk in Germany is estimated at 9.2–10.9%, meaning that on average one in 9–11 women will develop breast cancer during her lifetime [1, 3].

In view of the high incidence of breast cancer and the recognized fact that the disease requires multidisciplinary treatment, breast cancer management is prototypical of a complete process chain of care ranging from early detection, diagnosis and treatment through to follow up, the longest conceivable process chain of multidisciplinary care [7]. The diagnosis and treatment of breast cancer involves numerous interfaces for interaction and collaboration between medical specialties due to the need for multidisciplinarity and the bisectoral (in-patient vs. out-patient) nature of healthcare provision, especially in Germany. It was necessary therefore to centralize breast cancer services while in parallel creating a breast care network involving breast centres, breast units and doctors in private practice, including general practitioners, gynaecologists, medical oncologist and radiologists. The existing infrastructure in Germany is optimal and already provides the basis for such a network. There is a sufficient number of full-service university hospitals, district hospitals and regular and basic care hospitals that can collaborate with doctors in private practice to form a network to provide cancer care of the highest quality for all patients, whether they are covered by statutory or private health insurance.

While bisectoral care and multidisciplinary care both present considerable challenges, they also hold great potential for optimizing care. As breast cancer is prototypical of cancers that require multidisciplinary care, the disease probably represents the greatest challenge in terms of care optimization, but on the other hand also opens up many possibilities for health services research.

Modern oncology, which not only is based on the multidisciplinary diagnosis and treatment of malignancies but also is under the obligation to conduct quality assurance, faces the need to both optimize care and create transparency by introducing quality assurance procedures. The largest study, to our knowledge, in health services research and care optimization to date was therefore initiated to investigate these aspects of multidisciplinary oncology with the aim of evaluating and ultimately improving the quality of care on the basis of evidence-based medicine (EBM).

The nature of the problem outlined above made it necessary to iteratively develop a multi-step study design that would create the preconditions to: (1) define the interfaces along the process chain of breast cancer care, (2) standardize diagnostic and therapeutic procedures in a guideline-concordant manner, (3) analyse the importance of a multidisciplinary approach and (4) generate a body of EBM data, (5) enable the definition of standards for the centralization of breast cancer treatment and (6) review the quality of care, and (7) use these tools to certify breast centres.

To achieve these objectives, the following key questions were addressed:

- What is the rationale for centralizing diagnostic and therapeutic cancer services?
- Can quality indicators (QIs) be defined and used as key elements in a benchmarking programme designed to measure the quality of breast cancer care (“proof of principle”)?
- Is it possible to demonstrate that guideline concordance is achieved by implementation of a quality management system (QMS) designed to optimize structural, process and outcome quality; and can a network of quality assured and hence certifiable multidisciplinary breast centres be created at a national level (“proof of concept”)?

To address these questions the German Cancer Society (DKG) and German Society of Senology (DGS) jointly initiated the possibly largest multidisciplinary, multicentre cancer care research project, designed as a prospective interventional three-phase study (Table 1).

**Table 1:** Phases of the prospective interventional health services research study jointly initiated by the German Cancer Society and the German Society of Senology to achieve nationwide implementation of certified breast centres

Phase	Objective	Publication
Phase 1: benchmarking		
Phase 1a: proof of principle	To develop quality indicators based on the two relevant evidence-based, multidisciplinary national level-3 guidelines for breast cancer screening [23] and for the diagnosis and treatment of breast cancer in women [24]	Brucker et al. [35]. Benchmarking the quality of breast cancer care in a nationwide voluntary system: the first 5-year results (2003–2007) from Germany as a proof of concept
Phase 1b: analysis for a single specific specialty	To demonstrate the feasibility of subgroup analysis as illustrated by the example of radiation oncology	Brucker et al. [36]. Optimizing the quality of breast cancer care at certified German breast centres: a benchmarking analysis for 2003–2009 with a particular focus on the interdisciplinary specialty of radiation oncology
Phase 2: certification of breast centres (proof of concept)	To implement a quality management system to measure and assess structural, process and outcome quality	Brucker et al. [22]. Certification of breast centres in Germany: proof of concept for a prototypical example of quality assurance in multidisciplinary cancer care
Phase 3: nationwide implementation of certified breast centres	To combine voluntary provision of quality assurance data and external quality management auditing	Wallwiener et al. [37]. Zertifizierte multidisziplinäre Brustzentren. Ein Implementierungsprojekt der Deutschen Krebsgesellschaft und der Deutschen Gesellschaft für Senologie in Partnerschaft mit der Deutschen Gesellschaft für Gynäkologie und Geburtshilfe

In this review, we discuss the results of this study conducted in Germany and findings from other pertinent publications showing that it can now be considered an established fact that specialized team building and centralization of breast cancer care in certified breast cancer centres result in improved treatment—and, hence, improved long-term outcome—provided that quality assurance and QMS are defined and implemented, and the quality of care is quantitatively evaluated by benchmarking analysis. In addition, we also present the most recent data from the relevant programmes for voluntary benchmarking and certification of breast centres in Germany.

## Rationale for the creation of breast centres

Since 2003, a large body of data has been collected systematically from an increasing number of participating breast centres in Germany. As regards improvements in the quality of breast cancer care, the following key findings have been as follows:

- outcome improves with the number of treated breast cancer cases (centralization);
- the annual numbers of operations per centre and per surgeon (specialization) are important, and
- multidisciplinarity is of paramount importance.

These key points, which were open questions until 2003, have clearly provided the basis for the considerable improvements in the quality of breast cancer care which have since been achieved, as will be shown below.

### Why the creation of breast centres is important

The specific question as to the potential significant improvement in patient survival by centralization of breast cancer treatment in hospitals with a certain minimum annual volume was answered by, inter alia, the landmark study by Roohan et al. [8]. They analysed the 5-year survival rate and risk of death for 47,890 breast cancer surgery patients treated at 266 hospitals in New York State during 1984–1989 in relation to annual hospital volume, defined as the number of breast cancer surgeries per year. This analysis demonstrated a significant survival advantage for women treated surgically at centres with more than 150 breast cancer operations per year. Patients treated in hospitals with fewer than ten surgeries/year had a 60% increase in mortality risk, while the respective mortality risk for those treated in hospitals with 11–50 and 51–150 surgeries/year dropped to 30% and as little as 19%. More recently, a retrospective analysis by Guller et al. [9] of 233,247 patients with unilateral, localized primary breast cancer treated in the USA during 1988–2000 also found that high hospital volumes ≥70 cases/year were associated with better outcomes for breast-conserving therapy (BCT) and breast-ablative therapy (BAT). Compared with high-volume hospitals, low-volume hospitals with ≤30 cases/year had a statistically significant 3.04-fold increased risk of death after BCT and a significantly increased likelihood of postoperative complications after both BCT (risk-adjusted odds ratio (OR) 1.73) and BAT (OR 1.44). Length of stay was shorter and nonroutine discharge was lower at high-volume hospitals than at low-volume hospitals. Especially notable was the finding that the likelihood of receiving BCT was significantly higher at high-volume hospitals than at low- and intermediate-volume providers.

### Why specialization is important

It has long been established that overall survival increases with the specialization of the doctors involved in the diagnosis and treatment of breast cancer. For example, a study from Scotland investigated 5-year survival in 3,786 female breast cancer patients who underwent surgery between 1980 and 1988 and were followed up until 1993 [10]. This analysis compared treatment provided by specialist surgeons with treatment by nonspecialists. Multivariate analysis revealed increases by 9 and 8% in the 5- and 10-year survival rates, respectively, and a reduction in the risk of death by 16% in patients treated by specialists, regardless of age, socioeconomic status, tumour size, nodal status, or grading.

In their milestone publication, Gillis et al. [10] defined the term “specialist” as a surgeon with a special interest in the diagnosis and treatment of breast cancer characterized by the fact that treatment was carried out in a multidisciplinary breast centre, in collaboration with specialized surgeons, pathologists and oncologists there. In addition, the centre would also organize and conduct collaborative clinical studies and maintain separate records for all patients with breast cancer in their care.

### The role of the specialist surgeon

The connection between specialization and improved outcome is often attributed to optimized—i.e. guideline-compliant and individualized—adjuvant therapy, rather than surgeon experience. However, it is precisely this significant relationship between surgeon annual caseload and improved 5-year survival rate that was observed by Sainsbury et al. [11]. They showed that differences in survival rates existed independently of case mix (age, tumour stage at primary diagnosis and socioeconomic status) and could be explained by either surgeon caseload or treatment regime. Statistical significance was observed for a caseload >30 operations, compared with <10 operations per surgeon per year. By comparison, the EUSOMA guidelines [12], for example, recommend an annual caseload of at least 50 primary operations on newly diagnosed breast cancers per surgeon. However, Sainsbury et al. [11] also noted that quality depends crucially not only on minimum caseload but also on carrying out all treatment in a multidisciplinary setting.

Furthermore, details of clinical and pathological tumour stage and hormone receptors are more frequently available in the case of specialized surgeons [13]. Similarly, Golledge et al. [14] were able to demonstrate improvement in breast cancer survival rates after the advent of surgical subspecialization in Bedford, UK, in 1993. On the whole, before specialization, patients were seen by doctors who treated 10–38 new cases per year, whereas after the advent of specialization, diagnosis and treatment were performed only by doctors who saw 65–75 newly diagnosed breast cancer patients per year. Comparable tumour-node-metastasis (TNM) stages, patient populations and surgical procedures (equal percentage of BCT versus mastectomy) showed respective increases in 1- and 3-year disease-free survival rates from 87 to 91% and from 70 to 79%.

The improvement in outcome was also associated with a higher rate of axillary lymphadenectomy and the more frequent and more appropriate use of systemic chemotherapy and hormonal therapy, primarily tamoxifen, which only became possible with the advent of more accurate staging by axillary lymphadenectomy. At the same time, more attention was paid to obtaining a tumour-free surgical margin, which inter alia had a positive impact in terms of reducing local recurrence rate. Cady et al. [15] also emphasized the importance of the surgeon’s correct assessment of the tumour-free margin, on which the local recurrence rate depends.

Surgeon specialization and centralization can change not only the recurrence rate but also the proportion of BCT. A review by Grilli and co-workers [16] showed that better surgical management was offered, with more BCT and more appropriate indications for radical surgery/mastectomy. The rate of mastectomy relative to BCT was higher in smaller centres, even though there were no differences in tumour size and T1 tumours tended to be more frequent in smaller hospitals than in centres. McKee [17] and Kotwall [18] attributed this to, inter alia, the lack of multidisciplinary collaboration with the option of on-site radiation treatment.

The reduction of mortality or increase in 5-year survival can certainly also be attributed to adjuvant therapy, e.g. polychemotherapy or tamoxifen, which is more likely to be offered in a “high volume” hospital [8]. For example, the well-known meta-analysis of 133 randomized trials published by the Early Breast Cancer Trialists Collaborative Group showed relative improvements in overall survival of 28% for polychemotherapy and 25% for tamoxifen treatment [19].

Other studies reported similar results [10]. They emphasized that the observed survival benefit associated with treatment at a specialized centre was primarily the result of the more frequent and quality-assured administration of adjuvant systemic hormonal therapy, chemotherapy and radiotherapy, and combinations of these modalities. For example, although specialists perform axillary lymphadenectomies better and more frequently, a better prognosis is not only based on the surgeon’s experience but also on superior multidisciplinary organization and relevant experience in the provision of optimal adjuvant therapy. A more recent study by Kingsmore et al. [20] investigated the inter-relationship between adequacy of surgical management, locoregional recurrence and survival in 2,148 breast cancer patients treated with curative intent. This study from Scotland found that specialist treatment, after accounting for case mix and adjuvant therapies, was associated with a 57% reduction in 8-year local recurrence rates compared with nonspecialist treatment and that the risk of death from breast cancer was 20% lower. Kingsmore and colleagues concluded that the adequacy of surgical management was more frequent in specialist breast units, resulting in lower local and regional recurrence rates and correspondingly better survival rates.

### Why multidisciplinarity is important

All major studies emphasize the fundamental role that multidisciplinarity plays in improving patient survival. For instance, the well-known meta-analysis by Richards et al. [21] found that the 5-year survival rate was better when patients were treated in a multidisciplinary centre and the surgeon operated more than 30–50 new cases of breast cancer per year. Richards and colleagues went even further in their call for multidisciplinarity in supporting the recommendations of the Calman-Hine report [13] to implement a “hub and spoke” model. They showed that a region such as the West Midlands in the UK with a breast cancer incidence of 105/100,000 (about 5,250 new cases per year) required 16 cancer units, each feeding into one of four breast cancer centres. In this model, the “hub” is a central academic facility, a university hospital representing a “cancer centre” that conducts, implements, supervises and monitors basic research and the introduction of new treatments and conduct of clinical studies. Richards et al. considered it crucial to this model to introduce an information network to bind the centre and satellite units together and establish whether these structures lead to improvements in mortality and quality of life.

It is becoming increasingly clear from the literature, but only as far as breast cancer is concerned, that multidisciplinarity is more important than surgeon specialization. The surgical treatment of breast cancer, at least as far as mastectomy is concerned, is indeed less complex than surgery for colon or ovarian cancer. Therefore, most current discussions hypothesize that in colon and ovarian cancer it is surgeon caseload that is crucial whereas in breast cancer it is the caseload (and experience) of the expert team (radiologist, pathologist, surgeon, medical oncologist, and radiation oncologist) [22].

### The basis of quality assurance

Multidisciplinary care of patients with breast cancer requires a QMS with continuous quality assurance (QA), which includes comprehensive documentation and external analysis of the QA data. This is also a prerequisite for breast centre certification in accordance with the Requirements of Breast Centres (*Fachliche Anforderungen für Brustzentren*; FAB) developed by the DKG and the DGS. These requirements, in turn, are based on the two relevant evidence-based, multidisciplinary, national level 3 guidelines (S3-LL) for breast cancer screening in Germany [23] and for the diagnosis and treatment of breast cancer in women [24], both of which were jointly developed by the DGS, DKG and the relevant scientific medical societies. Quality of care in breast cancer is the focus of the joint collaboration between the DGS, the DKG and the German Society for Gynaecology and Obstetrics (DGGG) on the one hand and the West German Breast Centre (WBC), a subsidiary of the German Oncology Centre (DOC), on the other.

## Benchmarking quality of care, certification and nationwide implementation of breast centres

On the initiative of the DKG and DGS a large, nationwide, multidisciplinary, three-phase multicentre study (see Table 1) was initiated in 2003 to investigate strategies to improve the quality of cancer care by introducing voluntary benchmarking and certification programmes and implementing nationwide certification of breast centres. The results of these endeavours are reviewed in the following.

### Benchmarking the quality of breast cancer care

#### Proof of principle

Benchmarking is a continuous process aimed at systematically improving the quality of care. The benchmarking concept originates from economics, but can also be applied to hospitals and the treatments they offer [25]. Generally, hospitals will differ in terms of performance, showing good practices in some areas and scope for improvement in others. Various aspects of performance can be quantified by introducing indicators, the highest value for each indicator serving as the benchmark for that specific aspect of performance. In the present context, the aspects of performance pertain to the quality of breast cancer care and are represented by QIs. These can then be used to rank hospitals to identify the best performer for each QI. Competing with the best performers may unlock the other hospitals’ potentials for innovation by identifying “best practices”, modifying these practices appropriately and adopting them. Thus, each hospital can learn from the distinctive strengths of other benchmarking partners and specifically improve and expand its own service profile. If conducted anonymously, such benchmarking comparisons need not be a reason to fear, or risk, loss of prestige [26].

However, any nationwide benchmarking programme requires the development of an appropriate infrastructure to collect the necessary data in a standardized manner, calculate QIs according to uniform algorithms and perform a comparison. Specific quality objectives can be derived from operationalized clinical measures and be used as QIs to assess the quality of a breast centre and to analyse the changes in quality taking place over a defined observation period. QIs should represent all three types of quality that constitute a QMS, i.e. *structural quality* (e.g. number of staff and their qualifications, size of rooms, equipment), *process quality* (e.g. co-operation between specialist departments, communication flow, diagnostic and therapeutic parameters), and *outcome quality* (including complication and recurrence rates, disease-free survival and patient satisfaction) [26].

In the long term, the indicators of greatest interest in breast cancer care are those relating to outcome quality, i.e. morbidity and mortality. However, in breast cancer it often takes as long as 5–10 years for local recurrences and metastases to manifest. Breast cancer treatment generally extends over several years. Therefore it is necessary, at least temporarily, to resort to relevant short- and intermediate-term surrogate parameters to assess differences and improvements in quality over time [27–34]. In effect this means that during the first few years, the benchmarking programme mainly measures the extent to which the participating hospitals implement the guideline recommendations for diagnosis and treatment.

Little basic research has been done so far to investigate the impact of centralization and certification programmes in cancer care. To fill this gap, at least with regard to multidisciplinary breast centres, the first-ever prospective multicentre study investigating the implementation of a benchmarking programme at breast centres was conducted in Germany from 2003 to 2007 [35]. The study was subsequently extended until 2009 [36], and is still ongoing, the data for 2010 recently having become available [34].

Brucker et al. based their studies on a questionnaire which comprised 185 individual parameters derived from the DKG/DGS Requirements of Breast Centres (FAB) based on the relevant German level-3 guidelines [23, 24]. Specialist breast centres and hospitals with breast care units in Germany, Austria, Switzerland and the German-speaking parts of northern Italy participated in a benchmarking project on a voluntary basis. In Germany, a nationwide collaborative network of multidisciplinary breast centres was established and an external, independent organization, WBC, was commissioned by the DKG and DGS to collect and analyse the relevant data. A purpose-designed XML-based data set was developed and used for standardized data collection and calculations using uniform algorithms. A set of originally nine QIs was derived from guideline-based quality objectives, reviewed annually and developed further by modification or removal of existing QIs and the introduction of new QIs. Changes in QIs over time were analysed descriptively [35].

During the eight-year period from 2003 to 2010, the number of participating breast centres rose from initially 59 to 210, while the number of primary breast cancers as confirmed by postoperative histology increased from 5,994 to 34,678 (60% of approximately 58,000 new cases [1] per year in Germany). By 2010, the initial set of nine had increased to 18 QIs as surrogate indicators of long-term outcome quality. The 2003–2010 period saw marked increases for the following QIs: *preoperative histological confirmation of diagnosis* (QI 1; from 58 to 96%); *guideline*-*concordant endocrine therapy in hormone receptor*-*positive patients* (QI 6; from 27 to 97%); *guideline*-*concordant adjuvant and neoadjuvant chemotherapy* (*no age limit*) (QI 7.1b; from 32 to 78%); *radiotherapy after breast*-*conserving surgery* (QI 9a; from 20 to 87%); and *radiotherapy after mastectomy* (QI 10; from 8 to 74%) [34].

**Fig. 1:**
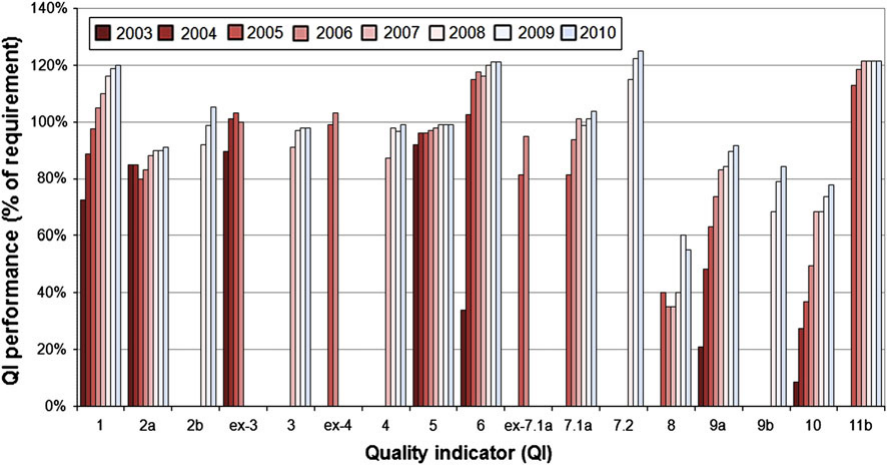
Relative performance of quality indicators (QIs) during the 2003–2010 period, expressed as a percentage of the respective DKG/DGS Requirements of Breast Centres (FAB) for the third year of certification (modified from [36] and updated according to [34]). QIs: *1* = preoperative histological confirmation of diagnosis, *2a* = appropriate axillary dissection, *2b* = patients with sentinel lymph node biopsy (SLNB), *ex-3* = complete tumour staging data, *3* = data on safety distance between tumour and resection margin, *ex-4* = HER 2/neu assessment, *4* = specimen imaging; *5* = hormone receptor assessment, *6* = guideline-concordant endocrine therapy in hormone receptor-positive patients, *ex-7.1a* = guideline-concordant adjuvant and neoadjuvant chemotherapy during the previous analysis period, age ≤70 years, *7.1a* = guideline-concordant adjuvant and neoadjuvant chemotherapy during the current analysis period, age ≤70 years, *7.2* = adjuvant combination chemotherapy with anthracyclines and/or taxanes, *8* = percentage of patients in clinical trials, *9a* = radiotherapy after breast-conserving surgery, *9b* = radiotherapy after breast-conserving surgery for ductal carcinoma in situ (DCIS), *10* = radiotherapy after mastectomy, *11b* = indication for breast-conserving therapy at T1

Figure 1 shows the changes in relative performance over time for all quality indicators for which the DKG/DGS Requirements of Breast Centres (FAB) specified performance levels for the third year of certification. Relative performance of each quality indicator is expressed as a percentage of the respective third-year requirement.

The DKG/DGS benchmarking programme thus allows detailed representation of the entire process chain of breast cancer care, both with regard to the situation at a particular moment in time and in terms of changes over time.

#### Subgroup analysis for a particular specialty

In addition to the overall benchmarking analysis, subgroups of relevant QIs can in principle also be used to demonstrate the extent to which progress has been achieved, or further improvement may still be needed, within a given specialty involved in the process. This was exemplified in a recent analysis of a subset of seven QIs of direct (QIs 9a, 9b, and 10) or indirect relevance to breast cancer radiotherapy [36]. The updated results of the subset analysis are summarized in Table 2, which shows that the QIs that directly reflect the guideline-concordance of radiotherapy (Nos. 9a, 9b, and 10) increased markedly over the study period. The percentage of patients given radiotherapy after breast-conserving surgery (QI 9a) or mastectomy (QI 10) increased from very low levels of 21 and 10% to high levels of 92 and 93%, respectively, relative to the third-year DKG/DGS minimum requirements of 95 and 80% for certified breast centres. QI 9b, which was newly introduced in 2008 to monitor radiotherapy after breast-conserving surgery (BCS) for ductal carcinoma in situ (DCIS), increased from 65 to 80%, equivalent to an increase in relative performance from 68 to 84% of the DKG/DGS minimum requirement (95% recommendation for radiotherapy after DCIS treated with BCS). QIs representing the availability of *complete tumour staging data* (No. ex-3), *data on safety distance* (No. 3), *intraoperative specimen imaging* (No. 4) indirectly relate to radiotherapy in that they reflect information that is important to the multidisciplinary tumour board when deciding on adjuvant therapy. These QIs also increased, though less markedly, over the study period.

**Table 2:** Quality indicators (QIs) of direct (9a, 9b, and 10) or indirect ([ex-3] to 8) relevance to radiation oncology and the 2003–2010 changes in their relative performance compared with the DKG/DGS requirements (modified and updated from [36] according to [34])

QI no.	Quality indicator (QI)	Tracked	2003	2004	2005	2006	2007	2008	2009	2010	Third-year DKG/DGS requirement (2010) (%)
[ex-3]	Complete tumour staging data	2003–2006	89%	101%	103%	100%	–	–	–	–	>95
3	Data on safety distance between tumour and resection margin	2007–2010	–	–	–	–	91%	97%	98%	98%	100
4	Specimen radiography (2007: preoperative in patients with microcalcifications; 2008: intraoperative)	2007–2010	–	–	–	–	87%	98%	97%	99%	>95
8	Percentage of patients in clinical trials	2005–2010	–	–	40%	35%	35%	40%	60%	55%	≥20
9a	Radiotherapy after breast-conserving surgery	2003–2010	21%	48%	63%	74%	83%	84%	89%	92%	>95
9b	Radiotherapy after breast-conserving surgery for DCIS	2008–2010	–	–	–	–	–	68%	79%	84%	>95^a^
10	Radiotherapy after mastectomy	2003–2010	10%	33%	44%	59%	81%	81%	88%	93%	>80

^a^Based on cases of BCS-treated primary DCIS with a recommendation for radiotherapy relative to the total number of cases of BCS-treated primary DCIS

Overall, the German voluntary programme for the external benchmarking of the quality of breast cancer care has produced remarkable results with respect to both breast cancer care in general and radiotherapy in particular. The programme has successfully documented the changes in breast cancer care which have taken place in Germany since 2003 and, in fact, been a driving force for quality improvement. The great acceptance of the benchmarking concept is also evidenced by the increase in case volumes at the participating DKG/DGS-certified breast centres in Germany (Table 3). For the first time valid evidence has been generated describing the reality of breast cancer care in Germany, and the collection of longitudinal follow-up data now appears firmly established.

**Table 3:** Certification of breast centres and case volumes at certified breast centres in Germany during 2004–2010

	31 Dec 2004	31 Dec 2005	31 Dec 2006	31 Dec 2007	31 Dec 2008	31 Dec 2009	31 Dec 2010
Certified breast centres	57	99	135	163	181	195	200
Certified sites	62	124	176	205	232	250	258
Applications under review	21	24	16	13	10	7	8
Sites per breast centre							
1	53	82	102	126	135	146	149
2	3	11	27	34	43	45	46
3	1	4	4	1	1	2	3
4	0	2	2	2	2	2	2
Primary breast cancers							
Total	11,152	20,089	27,722	33,955	41,322	48,289	52,345
Per breast centre	196	203	205	208	228	248	262
Per site	180	162	158	166	178	193	203
Percentage^a^	19.2%	34.7%	47.8%	58.6%	71.3%	83.3%	90.3%

^a^Relative to an estimated 57,970 primary breast cancers in Germany in 2006 [1]

### Certification of breast centres: proof of concept

The high incidence of breast cancer and the recognized need for the provision of appropriate, guideline-concordant multidisciplinary care make the management of breast cancer from early detection, diagnosis and treatment through to follow-up a prototypical example of a complete multidisciplinary and intersectoral process chain. This concept formed the starting point for, and was verified in, a prospective, iterative, interventional multicentre study conducted in Germany under the auspices of the DKG and DGS [22].

The certification project began with the first pilot certification of a breast centre to ISO 9001 and the DKG/DGS Requirements of Breast Centres (FAB) in December 2002. The DKG/DGS dual certification procedure in its present form was established in July 2003. Essentially it combines compliance with the FAB and the implementation and maintenance of a certified QMS at each individual centre.

Proof of concept was demonstrated when the first recertifications were achieved. At the end of 2005, the first two DKG/DGS-certified breast centres successfully achieved recertification after the initial 3-year certification. By mid-2008, 79 out of 80 breast centres had successfully completed the recertification process. One centre failed to meet the recertification requirements in 2007 and was therefore excluded from further participation in the DKG/DGS certification programme.

During the period from 2004 to 2010, as shown in Table 3, the number of certified breast centres increased from 57 to 200, with the number of single-site centres increasing from 53 to 149 and the number of two-site centres increasing from 3 to 46. Since 2006, however, the proportions of single-site and two-site centres have remained fairly constant at about 75 and 20–23%, respectively, as has the average number of sites per centre (about 1.3). In contrast, the proportion of multiple-site centres declined from 6.1 and 4.4% in 2005 and 2006, respectively, to 2.5% in 2010, indicating a consolidation trend towards single-site or two-site centres. During 2004–2010, the number of primary breast cancers treated at a certified breast centre increased 4.7-fold from 11,152 to 52,345 cases.

Over the 6-year period from 31 December 2004 until 31 December 2010, the number of new breast cancers per centre in the first year after certification increased 1.3-fold from 196 to 262, while the total number of primary breast cancers treated at breast centres with dual DKG/DGS certification increased 4.7-fold from 11,152 to 52,345 cases. Thus, in 2010, about 90% of the new cases of breast cancer in Germany, currently estimated at approx. 57,970 per year [1], were diagnosed and treated at a certified breast centre.

### Successful nationwide implementation of certified breast centres

As recently shown by Wallwiener et al. [37], these updated results confirm the findings of the unique descriptive study by Brucker et al. [22] demonstrating that voluntary certification of multidisciplinary breast centres according to the DKG/DGS dual certification procedure is well accepted in Germany. Moreover, Germany is now close to reaching the goals set by the European Parliament (EP) to create, by 2008, the conditions required to achieve reductions of 25% in average breast cancer mortality and of 5% in the disparity in 5-year survival between the countries of the European Union [38, 39]. Both EP resolutions also called for the creation of a network of certified multidisciplinary breast centres in accordance with the core criteria which the European Society of Breast Cancer Specialists (EUSOMA) published in 2000 and 2004 as *Requirements of a specialist breast unit* [12, 40], strongly advocating multidisciplinarity, specialization and centralization in the provision of cancer services. In 2005, Brucker et al. [7] estimated from calculations for the West Midlands in the UK [21] that in order to meet the EP targets Germany needed some 250 units and 63 large centres as proposed in the 1995 Calman-Hine report [13] (Table 4). With 200 breast centres certified by the end of 2010, Germany is now well on its way to the reaching the goal of creating a network of specialist breast units and certified breast centres according to the “hub and spoke” model of smaller units feeding into the large centres [21].

**Table 4:** Estimated number of breast units and breast centres needed in Germany to meet the European Parliament targets (from [7]), based on an extrapolation of the estimate for the West Midlands region of the UK [21]

Country/region	Annual new cases of breast cancer	Units	Centres	Units required by the EP
West Midlands (UK)	5,250^a^	16	4	16
Germany	44,274	250	63	250
Baden-Württemberg	5,673	32	8	32
Hesse	3,255	18	4–5	18
North Rhine-Westphalia	9,735	55	14	55
Schleswig–Holstein	1,506	9	2	9

^a^Based on an incidence of 105/100,000 in a population of approx. 5,000,000

## Extending the breast centre model to other cancers

Once proof of concept had been demonstrated for the certified breast centre, the next important question was to what extent this “prototype” of a certified multidisciplinary centre could also be applied to other cancers. Meanwhile, the DKG/DGS certification procedure has gained wide recognition in Germany as a general model for quality assurance in multidisciplinary cancer care. This has resulted in the creation of other site-specific and comprehensive cancer centres. Thus by the end of 2010, there were not only 200 DKG/DGS-certified breast centres but also 188 certified centres for colorectal cancer, 53 for gynaecological cancers, 63 for prostate cancer, 30 for skin cancer, and 18 for lung cancer in Germany. In addition, 11 comprehensive cancer centres had been created for a wide range of cancers, including pancreatic cancer and head-and-neck cancers.

## Summary and conclusions

Recent developments in German health policies reflect the increasing importance being attached to breast cancer. The primary aim is to co-ordinate and optimize breast cancer care in order to reduce the underprovision or overprovision of care by structured, intersectoral quality management (QM) [26, 35]. At first the shift in health policy thinking focused on the introduction of disease management programmes (DMPs) and early detection screening programmes, including statutory mammographic screening. The subsequent changes in clinical, scientific, public health and socioeconomic thinking necessarily led to a focus on breast cancer treatment and, consequently, to the called for improvements in the quality of care [41].

To reduce mortality, improve the quality of life and increase survival remains the common goal of all parties involved in the treatment of breast cancer. This requires quality assurance based on multidisciplinary, specialized management in a quality-assured, certified specialist unit which has a QMS in place and is regularly subjected to independent audits. The specialist breast units now need to be further integrated into a comprehensive, supraregional network within which care is provided according to (European) guidelines, studies are performed, data are collected from the network participants and uniformly documented, network-wide benchmarking is performed on the basis of uniformly defined QIs. The success of the implemented quality assurance measures is then assessed using well-structured documentation based on outcome quality and the performance of the individual QIs relative to pre-specified target values (DKG/DGS Requirements) [37].

As regards future conceptual orientation, the German scientific medical societies will have to continue working towards promoting quality in oncology both at the national and the European level. Intensive work in the *diagnostic area*, above all, has increasingly enabled women to receive primary treatment at an early stage, when prognosis is better. In addition, modern interventional techniques, especially minimally invasive procedures, to ascertain the diagnosis have contributed towards reducing delayed diagnosis and subsequent poorer prognosis. Similarly, thousands of unnecessary open biopsies can now be replaced by outpatient diagnostic interventional procedures every year [42]. The paradigm shift [43] in *therapeutic thinking* is based on reducing and adjusting the radicality of surgery to the requirements of the individual patient to preserve the breast whenever possible. This can be achieved using improved surgical techniques such as oncoplastic or reconstructive procedures [44], enabling a growing number of patients to have breast-conserving surgery while reducing the local recurrence rate due to histologically complete tumour resection [45].

In addition, *systemic tumour control* and *neoadjuvant and adjuvant therapy* [45] are increasingly being considered as treatment modalities. Thus, local tumour control has been integrated into multimodal systemic treatment strategies based on a definitive diagnosis by, e.g. diagnostic sentinel lymph node biopsy [46, 47] or the detection of disseminated tumour cells [48, 49].

In light of the existing and, in fact, increasingly emotionalized and politicized debate surrounding breast cancer it has apparently become inevitable to manage QA measures at the health policy level. Moreover, to improve the quality of care provided to breast cancer patients, Germany has recently passed legislation which for the first time links the provision of care to *minimum volume requirements*. Under Section 137 of Part Five of the German Social Code (SGB V), which governs statutory health insurance, the self-governing bodies within the German statutory health care system are obliged to determine minimum volumes for services where quality of outcome depends to a considerable degree on the volume of services provided. As of 2004, hospitals failing to meet these minimum volume requirements have not been authorized to provide such services. However, deviations from minimum volume requirements are permissible whenever nationwide provision of care is at risk.

On the subject of the legally required itemization of high-quality care, the SGB V stipulates that hospitals approved under Section 108 as well as prevention and rehabilitation facilities operating under a Section 111 contract are required to participate in quality assurance measures which must relate to the quality of treatment, medical care processes, and treatment outcomes, and must be designed to allow comparative assessment.

Ultimately it remains unclear, however, to what extent such nationwide standards of medical staffing, science-based care, technical equipment, and quality assurance by QMS implementation are affordable. In this context the study by Pagano et al. [50] still appears realistic, according to which the cost analysis for high-quality breast cancer centres with the appropriate specialization and multidisciplinary services indicates that an annual volume of at least 200 primary breast cancers appears favourable from an economic point of view. An additional factor that makes nationwide provision of care at this level appear very doubtful is the cost of permanent availability of multidisciplinary expertise and interaction. Beckmann et al. [51] analysed the cost-effectiveness of breast centres and pointed out that substantial portions of the costs of multidisciplinarity and centralization, including costs for the certification and re-certification, training and continuing education, research and documentation, did not qualify for reimbursement under the current reimbursement scheme in Germany, which is based on the diagnosis-related group system. They concluded that, under the current reimbursement conditions, certified breast centres could only exist as an integral part of a hospital where cross-subsidization from other departments can take place.

As regards the value of creating specialist and comprehensive centres and introducing certification, the conclusion is that despite the positive relationship between hospital annual case volume and surgeon annual caseload, and improvement in survival rates, these data do not necessarily always meet rigorous statistical criteria. It is clear, however, that multidisciplinarity and quality assurance are contributing decisively to improving cancer outcomes. For instance, a very recently published analysis of the clinical cancer registry data of 3,940 patients from the German region of Middle Franconia diagnosed with primary nonmetastatic breast cancer between June 2004 and March 2008 demonstrated that patients treated at certified breast centres were younger and had lower disease stages and lower grading [52]. The authors showed that, independently of the classical prognostic factors, the diagnosis and treatment services provided at certified breast centres improved the prognosis of breast cancer patients and attributed this to the quality-assured care based on the certification process. Overall, the introduction of quality assurance is also raising awareness of the processes involved in the provision of care and thus contributing to the improvement of multidisciplinary collaboration and, consequently, the improvement of patient care.

In addition to promoting the nationwide provision of care it is also necessary to promote the implementation of co-ordinating centres that support and supervise the transfer of the collective data to the benchmarking provider and the benchmarking analysis itself. These “centres of excellence” are essential prerequisites, especially with regard to knowledge transfer, study recruitment, scientific analysis and the implementation of current, up-to-date guidelines. This provides a basis from which the quality requirements developed by the German scientific medical societies can be harmonized with the health policies at the national and European levels.

Further information and details regarding, for example, the certification bodies, certification-related questionnaires and the DKG/DGS Requirements of Breast Centres are available online from the German Cancer Society (http://www.krebsgesellschaft.de) and the German Society of Senology (http://www.senologie.org).

Apart from the necessity to simplify the benchmarking procedure described above, enable cost-effective centralized procedures and reduce the bureaucracy of quality assurance and certification, the German statutory mammography screening programme should remain directly associated with the certified breast centres yet also involve the network of office-based specialists. Similarly, in view of the enormous and, what is more, unreimbursed amount of time and money spent on documentation, benchmarking and quality assurance, it is simply inconceivable that various parallel QA programmes can coexist without being harmonized. It should be stressed in this context that harmonization between national and international logical certification procedures has also not yet been implemented.

The fact that breast cancer has a high incidence and requires multidisciplinary care made this cancer a particularly suitable candidate for assessing whether the instrument of a nationwide quality-of-care benchmarking programme could serve as a prototype for the creation of cancer centres in general. In the future, however, the benchmarking procedure will need to be further standardized, though not only on the basis of the XML data set but also with regard to the competing programmes in Germany (DOC vs. BQS/AQUA), which need to be evaluated in a comparative manner and, if necessary, harmonized. Finally, the problem should be overcome that at least one-third of all German breast centres use their own benchmarking systems.

### Practical conclusions

The objective of establishing a Germany-wide network of certified multidisciplinary breast centres has largely been achieved. The next important step, the recertification of previously certified centres, which demonstrated proof of concept for the DKG/DGS certification programme, is well on its way and will require the introduction of even higher standards.

The implementation of certified multidisciplinary breast centres in the context of the efforts to optimize the quality of cancer care can be rightly considered an unparalleled success story which has also received growing international attention [22, 35]. Nonetheless, the breast services network still leaves scope for improvement along the entire process chain from mammographic screening, diagnostic interventions and treatment at a breast centre through to long-term follow-up.

At the present stage the true endpoints of breast cancer treatment, which include the long-term survival rate and the rates of recurrence and metastasis as indicators of outcome quality, can only be approximated by indicators of structural and process quality as surrogate endpoints. Even so, certified and uncertified breast centres tend to differ in respect of guideline-concordant treatment, also referred to as guideline compliance. Nevertheless, these differences can be expected to decrease as public awareness of these issues grows and the desirable turn to EBM as represented by the level-3 guidelines progresses. Thus it increasingly appears that developments in the quality of breast cancer care are reflecting the dictum that “the journey is the destination”.
